# No association between fruit or vegetable consumption and the risk of colorectal cancer in Japan

**DOI:** 10.1038/sj.bjc.6602566

**Published:** 2005-04-26

**Authors:** Y Tsubono, T Otani, M Kobayashi, S Yamamoto, T Sobue, S Tsugane

**Affiliations:** 1 Division of Health Policy, Tohoku University School of Public Policy, Kawauchi, Sendai 980-8576, Japan; 2 Epidemiology and Prevention Division, Research Center for Cancer Prevention and Screening, National Cancer Center, Tokyo 104-0045, Japan; 3 Statistics and Cancer Control Division, Research Center for Cancer Prevention and Screening, National Cancer Center, Tokyo 104-0045, Japan

**Keywords:** fruit, vegetable, colorectal cancer, prospective study, epidemiology

## Abstract

In a pooled analysis of two prospective studies with 88 658 Japanese men and women, fruit and vegetable consumptions, were not associated with a lower risk of colorectal cancer (705 cases); multivariate relative risk (95% confidence interval) for the highest *vs* the lowest quartile of intake being 0.92 (0.70–1.19) and 1.00 (0.79–1.27), respectively.

Although fruit and vegetables have been suggested to confer protection against colorectal cancer, recent prospective studies in Western populations found no or limited associations (Michels *et al*, 2000; Voorrips *et al*, 2000). In Japan, mortality from colorectal cancer increased during 1950–2000, especially in men (age-adjusted rate per 100 000 of 2.9–14.4 for colon and 5.6–9.3 for rectum in men; 3.3–9.5 for colon and 4.2–4.1 for rectum in women) (Statistics and Information Department, Minister's Secretariat, Ministry of Health, Labor, and Welfare of Japan, 2003). Dietary factors may play a part in this increase, but the role of fruit and vegetables remains unclear. We therefore examined the association between fruit and vegetable consumption and the risk of colorectal cancer in the Japan Public Health Center (JPHC) prospective study on cancer and cardiovascular disease.

## MATERIALS AND METHODS

The JPHC study has two population-based cohorts, and study designs are described in detail elsewhere (Otani *et al*, 2003). Briefly, Cohort I started in 1990 and included 40 106 subjects (19 345 men and 20 761 women) who were 40–59 years of age, lived in four Public Health Center districts, responded sufficiently to a self-administered questionnaire, and had no history of cancer (73.7% of the eligible subjects). Cohort II started in 1993 and included 48 552 subjects (23 180 men and 25 372 women) who were 40–69 years of age, lived in five Public Health Center districts, responded sufficiently to a self-administered questionnaire, and had no history of cancer (77.9% of the eligible subjects).

Cohort I questionnaire asked about the average consumption during the previous month of 44 food items including two fruit (fruit and fruit juice) and five vegetables (green leafy vegetables, yellow vegetables, white vegetables, pickled vegetables, and vegetable juice). Cohort II questionnaire asked about the average consumption during the previous month of 52 food items including three fruit (apples, oranges, and fruit juice) and six vegetables (green vegetables, carrot, tomatoes, green pickled vegetables, other pickled vegetables, and vegetable juice). The questionnaires had six frequency categories for fruit juice and vegetable juice that ranged from ‘rarely’ to ‘5 glasses day^−1^’, and four (Cohort I) or five (Cohort II) categories for other items that ranged from ‘never’ or ‘rarely’ to ‘almost everyday’. The amount of consumption of total fruit and total vegetables (g day^−1^) were calculated from these responses. We documented the questionnaire assessment of fruit and vegetable consumption to be reasonably valid (Kobayashi *et al*, 2002).

We followed up vital and residential status of subjects and incidence of cancer until the end of 1999. During 694 074 person-years of follow-up from the two cohorts, 705 cases of histologically confirmed colorectal cancer (456 colon and 249 rectum) were identified. Five percent of the subjects moved out of the study regions and 0.04% were lost to follow-up.

We used Cox's regression to compute from each cohort relative risk (RR) and 95% confidence interval (CI) of colorectal cancer according to quartiles of total fruit or vegetable consumption with adjustment for potential confounders. We pooled these estimates to obtain summary measures using inverse-variance weighting. As we observed no differential findings between the two cohorts, we present the pooled results only. This study has approximately 80% statistical power, with the two-sided *α*-error level of 5%, in detecting a true RR of 0.75 among the highest *vs* lowest quartiles of total vegetable consumption.

## RESULTS

Compared with men in Cohort I in the lowest quartile of total vegetable consumption, men in the highest quartile were more likely to engage in sports and use vitamin supplements, less likely to be current smokers, and consumed higher amount of meats and fish, but lower amount of cereals. The men in the two groups did not differ with respect to age, body mass index, or the prevalence of regular drinkers. We observed similar tendencies for women in Cohort I, and for men and women in Cohort II.

We found no significant association between fruit or vegetable intakes and the risk of colorectal cancer (Table 1). Multivariate RRs (95% CI) for the highest *vs* the lowest quartile of intake were 0.92(0.70–1.19) and 1.00(0.79–1.27), respectively, based on 705 cases. We observed no association whether or not colon and rectal cancers were separated, or men and women were separated. Exclusion of colorectal cancer cases diagnosed in the first 3 years of follow-up did not change the findings materially. Stratified analyses by covariates included in multivariate models did not reveal remarkable effect modifications. Analyses based on the octiles of total fruit or vegetable consumption did not show significant associations. No individual fruit or vegetables showed significant relations with risk.

## DISCUSSION

This is the first prospective cohort study of fruit and vegetable consumption and incident risk of colorectal cancer in Japan. Our results are consistent with the recent prospective studies in Western populations showing no substantial protective associations (Michels *et al*, 2000; Voorrips *et al*, 2000).

Our food frequency questionnaires had relatively small number of fruit and vegetable items and limited range of frequency categories. Nevertheless, we had observed in Cohort I an inverse association between fruit and vegetable intakes and the risk of gastric cancer (Kobayashi *et al*, 2002). It is therefore unlikely that failure to observe protective association was due to the crude designs of our questionnaires.

While mortality from colorectal cancer in Japan increased during 1950–2000, the average consumption of fruit and vegetables also increased during this period (42–117 and 242–311 g day^−1^, respectively) (Kenko Eiyo Joho Kenkyukai, 2002). Our results, along with these time trends, suggest that low consumption of fruit and vegetables is not primarily responsible for the increased rate of colorectal cancer in Japan.

## Figures and Tables

**Table 1:** Pooled multivariate RR and 95% CI of colorectal cancer for total fruit and total vegetable consumption^a^

	**Quartiles of total fruit consumption**					**Quartiles of total vegetable consumption**				
	**Lowest**	**Second**	**Third**	**Highest**	**Trend *P***	**Lowest**	**Second**	**Third**	**Highest**	**Trend *P***
Person-years in Cohort I	94 449	95 035	94 925	95 901		94 394	94 936	95 360	95 620	
Person-years in Cohort II	78 632	78 285	78 545	78 303		78 581	78 766	78 467	77 950	

*Men and women*										
Colorectum										
No. of cases	114/94	102/81	97/73	64/80		100/85	91/84	95/78	91/81	
RR (95% CI)	1.00	0.89	0.88	0.92 (0.70–1.19)	0.40	1.00	0.98	0.92	1.00 (0.79–1.27)	0.80
Colon										
No. of cases	77/56	70/51	66/48	43/45		67/50	60/53	68/44	61/53	
RR (95% CI)	1.00	0.89	0.93	0.92 (0.66–1.28)	0.61	1.00	0.99	0.96	1.08 (0.80–1.45)	0.73
Rectum										
No. of cases	37/38	32/30	31/25	21/35		33/35	31/31	27/34	30/28	
RR (95% CI)	1.00	0.88	0.78	0.91 (0.59–1.40)	0.47	1.00	0.95	0.84	0.87 (0.58–1.31)	0.37
										
*Men*										
Colorectum										
No. of cases	90/80	81/61	61/43	10/28		83/66	62/55	60/45	37/46	
RR (95% CI)	1.00	0.86	0.79	1.06 (0.70–1.61)	0.34	1.00	0.95	0.82	1.18 (0.88–1.59)	0.86
Colon										
No. of cases	59/51	57/36	42/31	8/16		57/40	42/36	41/27	26/31	
RR (95% CI)	1.00	0.83	0.86	1.02 (0.61–1.70)	0.57	1.00	0.96	0.84	1.24 (0.86–1.79)	0.69
Rectum										
No. of cases	31/29	24/25	19/12	2/12		26/26	20/19	19/18	11/15	
RR (95% CI)	1.00	0.91	0.68	1.19 (0.59–2.36)	0.42	1.00	0.91	0.81	1.06 (0.63–1.78)	0.81
										
*Women*										
Colorectum										
No. of cases	24/14	21/20	36/30	54/52		17/19	29/29	35/33	54/35	
RR (95% CI)	1.00	1.02	1.15	0.93 (0.61–1.42)	0.77	1.00	1.03	1.08	0.88 (0.57–1.35)	0.48
Colon										
No. of cases	18/5	13/15	24/17	35/29		10/10	18/17	27/17	35/22	
RR (95% CI)	1.00	1.07	1.19	0.87 (0.49–1.52)	0.86	1.00	1.09	1.25	1.01 (0.58–1.76)	0.96
Rectum										
No. of cases	6/9	8/5	12/13	19/23		7/9	11/12	8/16	19/13	
RR (95% CI)	1.00	0.77	0.95	0.84 (0.43–1.65)	0.77	1.00	0.96	0.84	0.71 (0.36–1.38)	0.27

RR=relative risk; CI=confidence interval.

a RRs have been adjusted for sex, age (5-year groups), Public Health Centre area, body mass index in kg m^−2^ (less than 19, 19–22.9, 23–26.9, and 27 or more), frequency of sports (never or 1 day/month or more), smoking (never, past, and current), alcohol consumption (non, occasional, 1–149, 150–299, and 300 g week or more), vitamin supplement use, quartiles of energy, cereals, meats, and fish by each cohort. The lowest quartile serves as reference category. The numbers of colon and rectal cancers are from Cohort I/Cohort II.

## Appendix A

The members of the Japan Public Health Center-based Prospective Study (JPHC Study) Group are as follows: S Tsugane, M Inoue, T Sobue, T Hanaoka, National Cancer Center, Tokyo; J Ogata, S Baba, T Mannami, A Okayama, National Cardiovascular Center, Suita; K Miyakawa, F Saito, A Koizumi, Y Sano, I Hashimoto, Iwate Prefectural Ninohe Public Health Center, Ninohe; Y Miyajima, N Suzuki, S Nagasawa, Y Furusugi, Akita Prefectural Yokote Public Health Center, Yokote; H Sanada, Y Hatayama, F Kobayashi, H Uchino, Y Shirai, T Kondo, R Sasaki, Y Watanabe, Nagano Prefectural Saku Public Health Center, Saku; Y Kishimoto, E Takara, T Fukuyama, M Kinjo, M Irei, Okinawa Prefectural Chubu Public Health Center, Okinawa; K Imoto, H Yazawa, T Seo, A Seiko, F Ito, Katsushika Public Health Center, Tokyo; A Murata, K Minato, K Motegi, T Fujieda, Ibaraki Prefectural Mito Public Health Center, Mito; K Matsui, T Abe, M Katagiri, Niigata Prefectural Kashiwazaki Public Health Center, Kashiwazaki; M Doi, A Terao, Y Ishikawa, Kochi Prefectural Chuo-higashi Public Health Center, Tosayamada; H Sueta, H Doi, M Urata, N Okamoto, F Ide, Nagasaki Prefectural Kamigoto Public Health Center, Arikawa; H Sakiyama, N Onga, H Takaesu, Okinawa Prefectural Miyako Public Health Center, Hirara; F Horii, I Asano, H Yamaguchi, K Aoki, S Maruyama, M Ichii, Osaka Prefectural Suita Public Health Center, Suita; S Matsushima, S Natsukawa, Saku General Hospital, Usuda; S Watanabe, M Akabane, Tokyo University of Agriculture, Tokyo; M Konishi, K Okada, Ehime University, Matsuyama; H Iso, Y Honda, Tsukuba University, Tsukuba; H Sugimura, Hamamatsu University, Hamamatsu; Y Tsubono, Tohoku University, Sendai; M Kabuto, National Institute for Environmental Studies, Tsukuba; S Tominaga, Aichi Cancer Center Research Institute, Nagoya; M Iida, W Ajiki, Osaka Medical Center for Cancer and Cardiovascular Disease, Osaka; S Sato, Osaka Medical Center for Health Science and Promotion, Osaka; N Yasuda, Kochi Medical School, Nankoku; S Kono, Kyushu University, Fukuoka; K Suzuki, Research Institute for Brain and Blood Vessels Akita, Akita; Y Takashima, Kyorin University, Mitaka; E Maruyama, Kobe University, Kobe; the late M Yamaguchi, Y Matsumura, S Sasaki, National Institute of Health and Nutrition, Tokyo; and T Kadowaki, Tokyo University, Tokyo, Japan.
